# Crosstalk of kidney and brain in diabetes-related cognitive impairment and therapeutic strategies

**DOI:** 10.3389/fendo.2025.1562518

**Published:** 2025-09-04

**Authors:** Chunmei Xu, Huikai Miao, Yongjun Chen, Lin Liao

**Affiliations:** ^1^ Research Institute of Acupuncture and Moxibustion, Shandong University of Traditional Chinese Medicine, Jinan, China; ^2^ Key Laboratory of Endocrine Glucose and Lipids Metabolism and Brain Aging, Ministry of Education, Jinan, China; ^3^ Department of Endocrinology, Shandong Provincial Hospital Affiliated to Shandong First Medical University, Jinan, China; ^4^ Shandong Key Laboratory of Endocrinology and Lipid Metabolism, Shandong Provincial Hospital, Jinan, China; ^5^ Department of Thoracic Surgery, Shandong Provincial Hospital Affiliated to Shandong First Medical University, Jinan, China; ^6^ South China Research Center for Acupuncture and Moxibustion, Clinical Medical College of Acupuncture, Moxibustion and Rehabilitation, Guangzhou University of Chinese Medicine, Guangzhou, China; ^7^ Department of Endocrinology and Metabology, The First Affiliated Hospital of Shandong First Medical University and Shandong Provincial Qianfoshan Hospital, Jinan, China; ^8^ Shandong Key Laboratory of Rheumatic Disease and Translational Medicine, Shandong Institute of Nephrology, Jinan, China

**Keywords:** diabetes mellitus, diabetic kidney disease, cognitive impairment, crosstalk, pharmacological therapy

## Abstract

Type 2 diabetes mellitus (T2DM) and its associated complications pose a global health threat. Notably, the rise in diabetes-induced cognitive dysfunction has garnered widespread attention. T2DM patients frequently face an elevated risk of both cognitive impairment and diabetic kidney disease (DKD) comorbidity. There is evidence to suggest that kidney and brain dysfunction share common pathogenic factors, such as vascular endothelial injury, oxidative stress, and inflammation activation. Whereas, the underlying mechanisms of kidney-brain interaction and effective treatments for DKD-related cognitive decline remain incompletely understood. Our review preliminarily summarized the relationship between renal dysfunction and cognitive decline based on the existing clinical trial evidence. The mechanisms underlying DKD-related cognitive decline which mainly included the accumulation of harmful metabolites, inflammation activation and endothelial dysfunction were also clarified. And the brain renin-angiotensin-aldosterone system (RAAS) may serve as a bridge connecting renal dysfunction in DKD with cognitive impairment. In addition, we further concluded that potential lifestyle interventions and pharmacological approaches, including antioxidants, RAS inhibitors, finerenone and hypoglycemic agents, such as sodium-glucose cotransporter-2 inhibitors, liraglutide and pioglitazone may exert the preservation of cognitive function. The review could offer valuable insights for therapeutic strategies of cognitive impairment associated with diabetes and DKD in future.

## 1 Introduction

**Figure 1:**
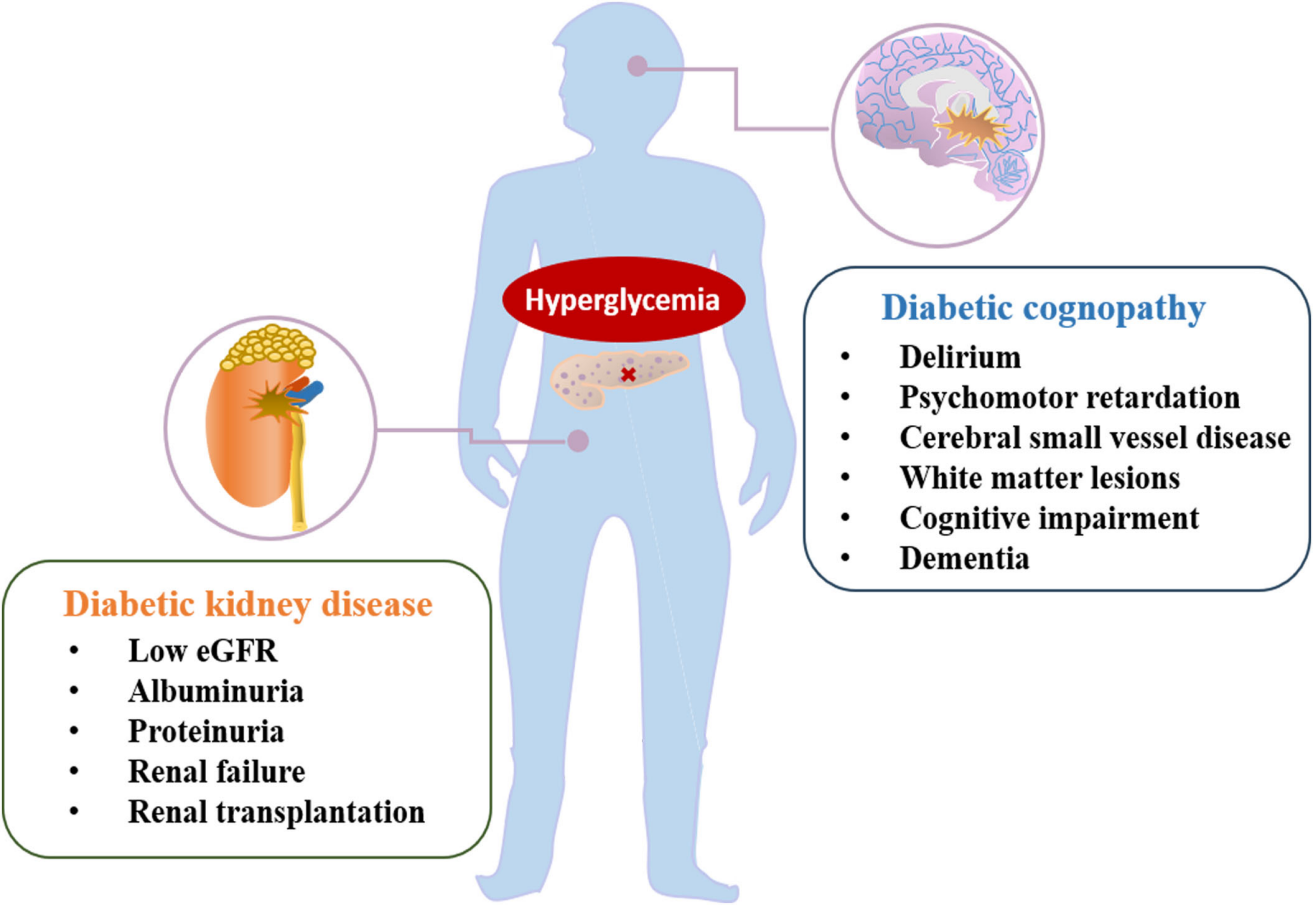
The main manifestation of diabetic complications, including diabetic kidney disease and diabetic cognopathy.

Diabetes mellitus (DM) is a group of endocrine and metabolic disorders characterized by hyperglycemia, and its incidence has been steadily increasing (1). Although advances in anti-hyperglycemic drugs have led to a decline in the incidence of diabetes and its complications over the past two decades, a significant number of DM patients still experience renal complications and an increased risk of renal failure (2). Cognitive impairment, another severe complication of DM, affects the brain function of diabetic patients and is a major risk factor for dementia. The number of people living with dementia continues to rise, making cognitive dysfunction and dementia a significant healthcare challenge, second only to stroke, with a high hospitalization rate and reduced quality of life for patients (3) (**Figure 1**). In recent years, cognitive dysfunction associated with type 2 diabetes mellitus (T2DM) has gained increasing attention and in-depth study (4, 5). T2DM is recognized as a risk factor for cognitive dysfunction and dementia (6). Furthermore, mounting evidence suggests that albuminuria and reduced estimated glomerular filtration rate (eGFR) are linked to declining cognitive function in individuals with diabetes (7–11). This, in turn, can contribute to the challenges in self-management of diabetic patients, exacerbating the risk of DM and its complications, including diabetic kidney disease (DKD) (12, 13). This indicates a complex, bidirectional relationship between DM and cognitive dysfunction. Currently, the progression of cognitive dysfunction in DKD patients and the underlying mechanisms remain to be fully understood. Therefore, it is crucial to elucidate the potential interplay among hyperglycemia, renal dysfunction, and cognitive impairment in diabetes patients for more precise future treatments.

## 2 Similarity between the kidney and brain

### 2.1 Both of kidney and brain share the similar endothelial damage under pathological condition

The kidney serves as a crucial center for endothelial signaling. Similar microvasculature structures in the kidney and brain may be connected to the pathogenesis linking renal impairment and cognitive dysfunction. Notably, both the renal and cerebral microcirculations share common characteristics of high flow and low resistance (14). Additionally, the arterioles in the brain bear a morphological resemblance to the juxtamedullary arterioles of the kidney, and both are responsible for maintaining vascular tone from parent vessels to capillaries (15). These shared characteristics make the vascular beds in both the brain and the kidney sensitive to blood pressure fluctuations (15). The biological action of blood flow auto-regulation is remarkably similar in both the kidneys and the brain. Auto-regulation of the cerebral and renal microvasculature ensures the supply of blood to end-organs while preventing excessive pressure exposure in the capillaries (14). Moreover, under pathological conditions, endothelial dysfunction leads to the loss of auto-regulation, exposing vascular beds to high pressure, causing protein leakage into the surrounding tissue and releasing inflammatory factors (16). Pathologically, the microvessels in the kidney of patients with albuminuria and cerebral microvasculature in patients with cognitive impairment share common characteristics, such as tortuosity and thickened basement membranes (17).

Indeed, the manifestations of cerebral small vessel disease (cSVD), including lacunar infarcts, white matter hyperintensities (WMH), and brain atrophy, bear similarities to diabetic microvascular complications, such as DKD (18). DKD is associated with progressive structural and functional network disorganization in the brain’s connectome, leading to the disruption of normally appearing structural-functional coupling (19). The mechanisms underpinning the associations between kidney injury and cognitive dysfunction include damage to microvascular circulation, resulting in hypoperfusion and blood pressure fluctuations, as well as increased inflammation and oxidative stress in in both the brain and kidneys. Endothelial loss in the brain may contribute to the accumulation of amyloid and disrupted transport (20), increasing susceptibility to silent brain infarcts (SBIs) and white matter lesions (WMLs) (21). Similarly, endothelial dysfunction in the kidney is manifested by elevated levels of albuminuria, defects in the filtration barrier, the initiation of tubulointerstitial inflammation, and subsequent renal fibrosis (22). Thus, albuminuria may serve as an early indicator of damage to cerebral arteries. Elevated systolic blood pressure and older age are critical factors associated with a decline in cognitive function in conjunction with albuminuria. Furthermore, elevated inflammation factors, metabolic disturbances-related indicators, increased arterial stiffness (23), and the influences of obesity (24) appear to contribute to albuminuria-associated cognitive dysfunction. Additionally, a randomization study conducted by Marini and colleagues showed that increased urinary albumin creatinine ratio (UACR) and decreased eGFR were implicated in large artery stroke, suggesting that cerebral arterial disease shares a common genetic mechanistic basis with kidney disease (25).

### 2.2 Renin-angiotensin-aldosterone system and cognitive impairment

The brain renin-angiotensin-aldosterone system (RAAS) plays a vital role in neural function homeostasis and normal cognitive function, including memory, consolidation, and information retrieval (26). However, dysfunction in the brain RAAS is a critical factor contributing to cognitive impairment and dysfunction (27–29). When RAAS is hyperactivated, it leads to various effects, including cerebrovascular remodeling, vascular inflammation, accumulation of reactive oxygen species (ROS), increased synthesis and secretion of aldosterone, and astrocyte senescence. These effects further contribute to neurodegeneration and impaired cognitive function.

**Figure 2:**
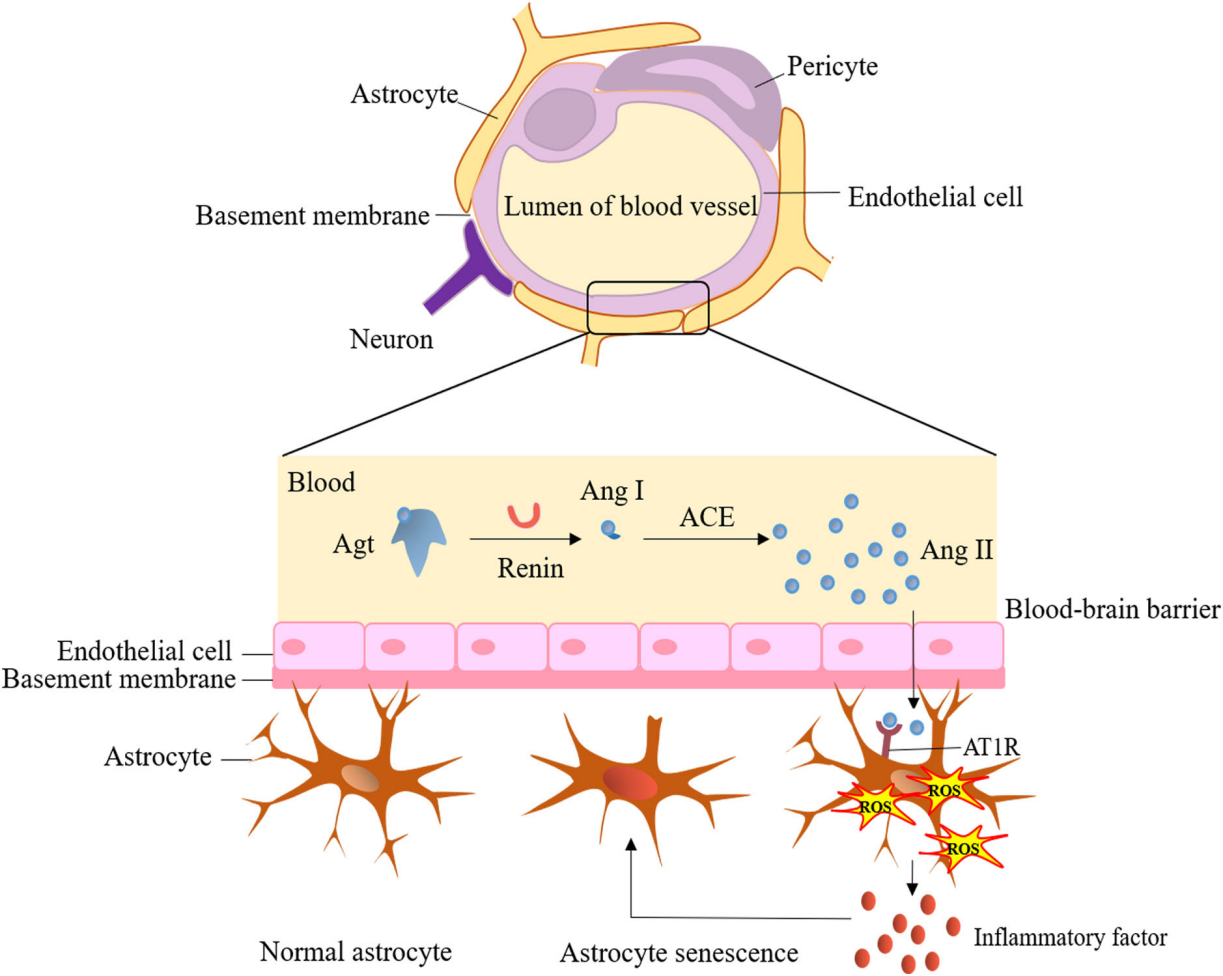
Hyperactivation of brain renin-angiotensin-aldosterone system and cerebral dysfunction. ACE, angiotensin-converting enzyme; Agt, angiotensinogen; Ang I, angiotensin I; Ang II, angiotensin II; AT1R, angiotensin II type 1 receptor; ROS, reactive oxygen species.

Notably, enhanced activation of angiotensin II type 1 receptor by angiotensin II and the mineralocorticoid receptor by glucocorticoids and aldosterone can increase blood-brain barrier (BBB) permeability, induce oxidative stress, neuroinflammation, and astrocyte dysfunction (**Figure 2**), while also decreasing cerebral blood flow (28). Indeed, angiotensin receptor blockers (ARBs) have shown a significant reduction in dementia and the progression of Alzheimer’s disease compared to other antihypertensive treatments (30), highlighting the significant relationship between RAAS activation and cognitive decline. It is well-known that RAAS activation is responsible for renal damage in DM patients, and inhibiting RAAS can effectively delay the progression of DKD. Therefore, RAAS may serve as a bridge connecting renal dysfunction in DKD with cognitive impairment, and its role and underlying mechanisms warrant further exploration.

## 3 The relationship between renal dysfunction and cognitive impairment

### 3.1 Correlation of renal damage with cognitive decline

Chronic renal injury is primarily characterized by albuminuria and/or a low eGFR, and it is strongly linked to diabetes (31–33). A systematic review has indicated that advanced chronic kidney disease (CKD) is associated with cognitive impairment as observed through cerebral magnetic resonance imaging (MRI) technology, transcending ethnic background (34). Moreover, numerous studies have reported a higher prevalence of cognitive impairment among CKD individuals compared to the general population (35, 36). The Intervention Project on Cerebrovascular Diseases and Dementia in the community of Ebersberg (INVADE) study reported a cognitive decline prevalence of 21% in CKD patients (37). Longitudinal investigations have also revealed a two-fold increased risk of cognitive impairment in CKD patients compared to the general population (38).

Despite variations in methodologies and outcome assessments, several studies have established associations between albuminuria and the risk of developing cognitive impairment and dementia, as well as correlations with imaging markers for cognitive decline. A recent meta-analysis has also confirmed these associations based on results from population-based studies (39). Gabin et al. (40) observed a positive association between microalbuminuria and various subtypes of dementia, including Alzheimer’s disease, vascular dementia, a mixture of Alzheimer’s disease and vascular dementia, dementia with Lewy bodies, and frontotemporal dementia. The ONTARGET/TRANSCEND studies have shown that albuminuria-related factors contribute to cognitive function decline, and the progression of albuminuria is closely linked to cognitive dysfunction (8) Data from the Cardiovascular Health Study revealed a significantly increased risk of cognitive decline as the UACR increased (41). A substantial body of evidence supports the correlation between albuminuria and executive function. The Nutrition, Aging, and Memory in Elders (NAME) Study, which includes older subjects from various racial backgrounds, found that microalbuminuria was associated with poor executive performance (11). Furthermore, another study (7) noted that the level of albuminuria was related to executive dysfunction.

Several prospective studies have also supported the idea that associations exist between albuminuria and eGFR with cognitive decline in CKD patients. The Rancho Bernardo Study revealed that baseline microalbuminuria was significantly associated with cognitive functional decline over a 6-year follow-up period (42). Individuals with elevated urinary albumin levels exhibited a significantly higher risk of cognitive decline during the follow-up (23, 42, 43). Findings from the Third National Health and Nutrition Examination Survey (NHANES III) demonstrated that moderately decreased eGFR levels in individuals were correlated with poor cognitive performance, including deficits in visual attention, learning, and concentration (44). A longitudinal study investigating mediators related to cerebral atrophy demonstrated that increases in albuminuria and low eGFR levels served as independent risk factors for hippocampal atrophy, which is associated with word recall function. Consequently, effective management of renal impairment may contribute to preserving brain atrophy and cognitive decline (45).

End-stage renal disease (ESRD) is a leading cause of renal injury, necessitating renal replacement therapy. Patients with ESRD are prone to encephalopathy and cognitive impairment due to the accumulation of uremic toxins. Cognitive dysfunction in these individuals is characterized by confusion, sleep disturbances, and, in severe cases, deep coma (46). A British cohort investigation indicated that around 68% of patients with ESRD experienced cognitive impairment (47). Furthermore, the Brain in Kidney Disease (BRINK) cohort study reported that patients with more advanced stages of CKD were more likely to develop cognitive impairment (48), suggesting that the risk of cognitive dysfunction increases with the severity of renal injury in CKD patients. However, certain meta-analyses have reported no clear association between impaired kidney function and cognitive function, regardless of the stage of CKD (38, 49), indicating that cognitive impairment occurs with a similar frequency across all CKD stages.

Hemodialytic therapy is considered a major contributor to chronic dialysis encephalopathy (50). Studies have revealed a high prevalence of cognitive dysfunction in ESRD patients after chronic dialysis (51, 52). Up to 70% of patients undergoing hemodialysis and 60% of those on peritoneal dialysis have developed moderate to severe cognitive dysfunction (53, 54). Another multicenter cross-sectional study has shown that patients with ESRD receiving peritoneal dialysis are at a significantly increased risk of cognitive dysfunction and abnormal immediate memory and executive function (55). In ESRD patients undergoing dialysis, cerebral perfusion flow impairment is common (56, 57), particularly in hemodialysis. Cerebral perfusion declines contribute to vascular damage, abnormalities in cerebral structure (58, 59), and the presence of cerebral WMLs (60), ultimately leading to brain dysfunction characterized by impaired processing speed and executive function.

Furthermore, a cross-sectional study has demonstrated that higher levels of mineral metabolism and fibroblast growth factor 23 (FGF-23), which have been correlated with adverse cardiovascular events and death (61) in patients receiving hemodialysis are independently associated with worse memory scores in comprehensive neurocognitive tests (62). This suggests that FGF-23 may act as an independent contributor to cognitive impairment in ESRD patients. Notably, substantial evidence indicates that cognitive impairment in patients with kidney dysfunction can be partially reversed with dialysis (63) and kidney transplantation (64). Another clinical study revealed that patients treated with nocturnal hemodialysis experienced a significant improvement in attention, processing speed, psychomotor efficiency, and working memory (65). However, no significant changes were observed in the domain of learning efficiency, implying that hemodialysis may primarily benefit the amelioration of early changes in thought processing rather than memory improvement.

### 3.2 Mechanisms of cognitive decline associated with renal injury

**Figure 3:**
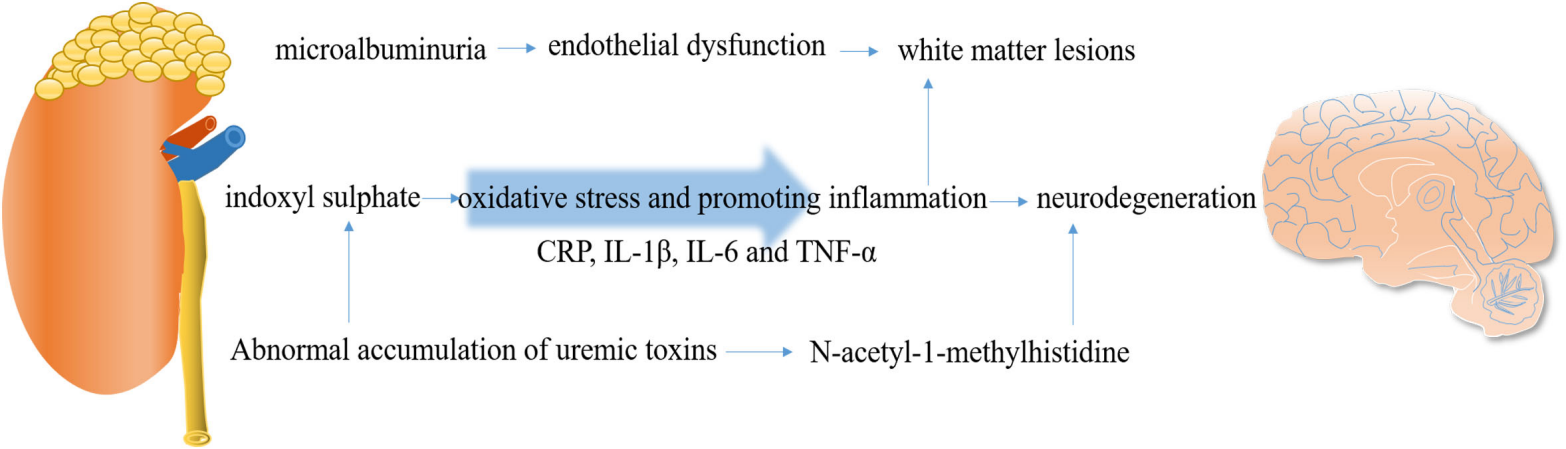
Crosstalk between the kidney and brain and potential mechanisms.

The causes of cognitive impairment in CKD are multifactorial and include cerebrovascular disease, WMLs, endothelial dysfunction, renal anemia, dialysis disequilibrium, and uremic toxin accumulation (66). The relative mechanisms are summarized in **Figure 3**.

#### 3.2.1 Endothelial damage

Endothelial integrity is crucial for maintaining the function of the BBB. Damage to the endothelium can initiate BBB breakdown, contributing to the development of cSVD (67). The neurovascular unit, composed of brain’s indigenous cells such as endothelial cells, neurons, microglia, and astrocytes, is essential for maintaining brain physiology and BBB integrity (68) (**Figure 2**). Microalbuminuria, a common early marker of CKD, has been emphasized as a significant indicator, as it can reflect abnormal vascular endothelial function. The HOORN study conducted by Murea et al. demonstrated that microalbuminuria is associated with maladaptive arterial remodeling and impaired arterial flow-mediated vasodilation due to endothelial dysfunction (69). Damage to the endothelial glycocalyx can lead to increased capillary wall permeability, resulting in albuminuria (70) and impaired cerebral and coronary microcirculations (71). Data from a cross-sectional study revealed that the accumulation of advanced glycation end products (AGEs) can disrupt vascular endothelial function (72). Under the conditions of ESRD, proinflammatory factors can also decrease cerebral blood flow and induce endothelial dysfunction, further promoting the occurrence of cerebrovascular disease and cognitive impairment. Microalbuminuria is associated with brain atrophy, and this phenomenon may be explained by the shared pathophysiology between the kidneys and the brain. It is proposed that microalbuminuria reflects renal expression of systemic endothelial dysfunction. Gradual endothelial damage allows serum proteins to extravasate into brain extracellular spaces, contributing to brain abnormalities observed in MRI, primarily an increase in WMH. Therefore, microalbuminuria in CKD patients may mediate the increase in WMH, leading to cortical thinning and brain atrophy, ultimately resulting in cognitive impairment (73). Another marker of endothelial dysfunction, UACR, which reflects systemic damage in vascular microcirculation, is also linked to an increased burden on cerebral vessels and cognitive dysfunction (74).

#### 3.2.2 White matter lesions

CKD has been associated with structural brain changes, particularly WMLs (75, 76). Associations between albuminuria and low gray matter (GM) volume, high cerebrospinal fluid (CSF) volume indicative of cerebral atrophy, and a higher WM disease burden have also been observed. The effects of albuminuria on increased WML volume and decreased GM volume may be mediated by systemic endothelial dysfunction in early CKD (77).

#### 3.2.3 Uremic toxins

Uremic toxins are believed to be the primary cause of cognitive impairment in CKD patients (78). However, the exact role and mechanism of uremic toxins in cognitive disorders have not yet been determined. A longitudinal population-based cohort study reported that patients with ESRD exhibit severe frailty and demonstrate cortical area-specific alterations in energy (79). Uremic toxins, such as indoxyl sulfate, impair glial cell function by enhancing oxidative stress and promoting neuroinflammation, providing a foundation for subsequent neurodegeneration (79). Indoxyl sulfate, a protein-bound uremic toxin, is considered one of the most important uremic toxins and is known to cause nephrotoxicity, especially in tubular cells, as well as the suppression of endothelial cell proliferation and the induction of increased free radicals (80). Importantly, it is not completely eliminated by dialysis. Indoxyl sulfate has been directly implicated in the pathways underlying abnormal oxidative stress through which CKD affects cognitive function. This includes the upregulation of NADPH oxidase and glutathione levels, activation of NF-κB, alteration in glial cell proliferation, and impaired antioxidant response mediated by nuclear factor erythroid 2-related factor 2 (Nrf2) (63).

#### 3.2.4 Pro-inflammatory factors

Appropriate activation of the inflammatory response is essential for defense against injuries, but if not properly regulated, deleterious effects may occur (81). Inflammation plays a crucial role in neurodegenerative and vascular diseases by promoting platelet aggregation and atherogenesis (82). A significant inflammatory response is observed in patients with T2DM and cognitive dysfunction, and the synergistic effect between peripheral and central inflammation might lead to the occurrence of diabetic cognopathy (DC) (83). Several inflammatory biomarkers such as C-reactive protein (CRP), interleukin-1β (IL-1β), interleukin-6 (IL-6), and tumor necrosis factor-α (TNF-α) have been confirmed to be correlated with SBIs, WMH, decreased eGFR, and a high UACR (84, 85). This indicates that common inflammatory factors are implicated in the occurrence of cSVD and CKD. Therefore, it is reasonable to assume that a parallel process of inflammation exists in both the brain and kidneys, which may mediate CKD-related cognitive decline.

Under normal circumstances, peripheral tissues and the brain are separated from each other due to the intact blood BBB. However, under pathological conditions, such as in T2DM, the BBB is compromised, leading to the abnormal activation of AGE and the receptor for AGE (RAGE) interaction, setting off a self-perpetuating cascade (83). When the BBB loses its integrity, various chemokines and cytokines, including IL-1β, IL-6, TNF-α, and macrophage colony-stimulating factor, may enter the functional areas of the brain, inducing cerebral damage and cognitive decline. Moreover, increased AGEs can pass into peripheral tissues, activating RAGE, which generates ROS and activates NF-κB signaling in both peripheral tissues and the brain, further disrupting the BBB and forming a vicious cycle. The AGE/RAGE interaction is believed to play a central role in the inflammatory synergy between T2DM and DC, negatively impacting cognitive function. As renal function worsens, neurodegenerative diseases exacerbate due to the cumulative effects of inflammation and endothelial dysfunction. Further clinical evidence is required to clarify the impact of pro-inflammatory factors on cognitive dysfunction.

#### 3.2.5 Harmful metabolite accumulation

Metabolomics is increasingly applied to explore novel biomarkers and mechanisms of CKD and cognitive decline. In the Atherosclerosis Risk in Communities (ARIC) cohort, a histidine metabolite, N-acetyl-1-methylhistidine, was remarkably associated with verbal memory in African Americans (86), suggesting that the abnormal accumulation of harmful metabolites is responsible for cognitive impairment.

## 4 Overview of diabetes-related cognitive impairment

Accumulating evidence has clarified the association between diabetes and an increased incidence of cognitive dysfunction. In patients with DM, central nervous system dysfunction related to hyperglycemia is common (87). Diffuse changes in the central nervous system and generalized lesions in neurons and axons have been observed in patients with DM (88). Additionally, studies have reported that the severity of cerebral function decline is correlated with the duration of exposure to hyperglycemia, as determined through neuropsychological tests (87). Decreases in cerebral cortex volume and loss of neocortical neurons have also been observed in experimental mice with hyperglycemia induced by streptozotocin (89). These experimental findings strongly suggest a potential correlation between diabetes and cognitive impairment.

Although various factors, including chronic hyperglycemic stimuli, repeated episodic hypoglycemia and ketoacidosis, damage to the BBB, insulin dysfunction in the brain, cardiovascular risk factors, and metabolic changes, have been explored, the pathogenesis of diabetes-related cognitive impairment is still a subject of preliminary investigation. The potential mechanisms of DM-related cognitive decline are summarized in **Table 1**.

**Table 1:** A summary of research mechanism of diabetes-related cognitive dysfunction.

Pathogenic factor	Role in the cognitive decline	Regulatory mechanism	Source	Reference
Cerebral structural lesions	Gray matter, white matter, and hippocampal volume loss, and WMLs in diabetes and DKD-related cognitive impairment	Regional cerebral volume loss and hippocampal dysfunction	An observational study	(90)
Insulin homoeostasis disorder	Decreased insulin levels	Accumulation of circulating and cerebral β-amyloid peptides	An experimental study using diabetic rats	(91)
Hippo signaling activation	Diabetes-related cognitive impairment	Maintaining glucose homeostasis and regulating peripheral insulin pathway	Streptozotocin-induced diabetic rats	(92)
Decrease of N-acetyl aspartate/creatine	Hyperglycemia-related cognitive decline	Metabolic abnormalities in the brain	A perspective study	(93)
Mutual interaction of inflammation activation and endothelial dysfunction	DKD-related cognitive decline	Enhanced oxidative stress, inflammatory factors, and sorbitol production	Clinical studies	(25, 94)
Increase of nitric oxide inhibitor	Renal damage-related cognitive impairment	Disrupting the integrity of BBB, contributing to the injury of cerebral vascular endothelium	An experimental study using CX3CR1 deficiency mice	(95)
Impaired clearance of β-amyloid peptide	Development of cognitive decline under the condition of renal dysfunction	Accumulation of amyloid plaques in the brain	A systematic analysis	(96)
Decrease of left N-acetyl-aspartate and increase of left choline level	Cognitive decline in DKD patients	Increased cholinesterase activity, cholinergic nerve cell dysfunction and disruption of integrity	A retrospective study	(97)
Low eGFR level	Cognitive performance related with renal impairment	Low concentration of plasma homocysteine	A cross-sectional study	(98)
Retinol-binding protein 4	Mediator of cognitive dysfunction for DKD patients with silent cerebral infarction	Upregulation of Lp-PLA2 and Netrin-1	An observational study	(99)

BBB, blood-brain barrier; CX3CR1, C-X3-C motif chemokine receptor 1; DKD, diabetic kidney disease; eGFR, estimated glomerular filtration rate; Lp-PLA2, lipoprotein-associated phospholipase A2; WMLs, white matter lesions.

### 4.1 Loss of gray matter, white matter, and hippocampal volume

The loss of GM, WM, and hippocampal volume, as well as WMLs, is believed to underlie DM-associated cognitive dysfunction (100). MRI studies in individuals with T2DM have reported diabetes-related decreases in total GM and WM volume, as well as regional patterns of cerebral volume loss, including hippocampal involvement (101, 102). The hippocampus is crucial for memory function and is particularly susceptible to neurotoxic stimuli associated with CKD and T2DM, including chronic hypoperfusion and hypoglycemia (103). An observational study reported a direct relationship between T2DM and the pathological manifestations of Alzheimer’s disease, including similar patterns of regional cerebral volume loss and hippocampal dysfunction (90).

### 4.2 Insulin homeostasis disorder

The potential role of disordered insulin homeostasis in cognitive impairment has been clarified. Studies have shown that insulin can regulate cognition by interacting with insulin-degrading enzyme, which is involved in cerebral tau metabolism (104). An experimental study found that decreased insulin levels in diabetic rats could lead to the accumulation of circulating and cerebral Aβ peptides, contributing to cognitive dysfunction in Alzheimer’s disease (91). These studies provide preliminary evidence of the mechanisms by which insulin modulates cerebral function in patients with T2DM.

### 4.3 Abnormal activation of Hippo pathway and amyloid polypeptide accumulation

Yu et al. conducted an experimental study and discovered that changes in the Hippo signaling pathway contribute to cognitive decline in streptozotocin-induced diabetic rats. This finding suggests that therapeutic interventions modulating the Hippo signaling pathway may hold the potential to ameliorate diabetes-related cognitive impairment (92). T2DM and Alzheimer’s disease, a leading cause of cognitive impairment, share common characteristics such as genetic predisposition, advanced aging, and a parallel pathological accumulation of amyloid in both pancreatic islets and the brain (105). Consequently, it is plausible that a common underlying mechanism involving the deposition of cerebral amyloid in islets may contribute to diabetes-related cognitive dysfunction.

### 4.4 Decrease of N-acetylaspartate/creatine

Hyperglycemia plays a crucial role in the onset of early cognitive decline by inducing reductions in myelination and the decrease in the cerebral content of N-acetyl aspartate (NAA) and creatine ratio, a significant marker of neuronal and axonal loss (106). Furthermore, the association between glycosylated hemoglobin (HbA1c) and the NAA/creatine ratio suggests a potential correlation between overall glucose control and metabolic abnormalities in the brain (93). These findings imply that the reduction in the NAA/creatine ratio may be implicated in the development of diabetes-related cognitive decline.

## 5 Kidney-brain interaction under the hyperglycemic condition and underlying mechanisms

Studies examining the relationship between cognitive impairment and clinical microvascular complications suggest that a “microvascular” pathway may contribute to cerebral dysfunction as a consequence of microangiopathy (107). Diabetic patients with renal injury exhibit significant disruptions in both structural and functional brain networks, as well as alterations in structural-functional coupling, particularly in comparison to diabetic patients without kidney dysfunction. This disruption may worsen with increasing renal dysfunction. Although the underlying mechanisms remain poorly understood, these observations emphasize the pivotal role of the kidney-brain axis in neurocognitive dysfunction. The degree of microvascular complications across multiple organ systems may influence this relationship.

### 5.1 Relationship between DKD renal dysfunction and cognitive decline

DKD can serve as a risk factor for the incidence of cerebral infarction. Under conditions of abnormal glucose metabolism, elevated Ca^2+^ concentrations and erythrocyte aggregation can damage cell membranes. This leads to hypoxia, the release of various active substances, swelling of endothelial cells, increased nerve cell apoptosis, and even the formation of cerebral infarctions. However, conclusive evidence regarding the direct relationship between DKD and cognitive decline is still lacking. Research has indicated that mild cognitive impairment and dementia are common in DM patients who develop albuminuria (108). Conversely, a cross-sectional study named the Reasons for Geographic and Racial Differences in Stroke (REGARDS) study reported little influence of T2DM on the association between severe renal dysfunction and cognitive impairment (109). For middle-aged T2DM patients (110), there is evidence of an independent and negative correlation between albuminuria and cognitive function, even with a short duration of less than 5 years for the onset of cognitive dysfunction. This may be due to a possible contribution of albuminuria at the early stages of cognitive deterioration. Another study also revealed that 48% of patients with stage 3 or 4 DKD had cognitive dysfunction and neurocognitive disorders (111). Liao et al. (55) reported that 35% of DM patients undergoing peritoneal dialysis experienced cognitive impairment, and the cognitive function of these patients was comparable to that of non-diabetic individuals. Therefore, the comorbidity of diabetes and renal dysfunction significantly contributes to cognitive impairment. Additionally, the relationship between increased HbA1c levels and cognitive dysfunction in DKD has been well-established. Seidel et al. (112) clarified that HbA1c is associated with cognitive impairment in patients with renal dysfunction. Another study also demonstrated that an increased concentration of HbA1c is linked to cognitive impairment in diabetes (113).

The role of post-transplantation dementia in individuals with diabetic ESRD in relation to cognitive dysfunction remains controversial. Fiorina et al. conducted a study and verified that, in contrast to healthy subjects, a higher incidence of cerebrovascular disease is observed in patients with ESRD combined with type 1 diabetes mellitus (T1DM) (93). Psychological and neuropsychological function measurements indicated better scores for ESRD plus T1DM subjects after kidney-pancreas transplantation, suggesting a potential therapeutic effect of improving kidney function on cognitive impairment. Diabetes combined with ESRD is associated with cerebrovascular disease and cognitive decline. However, a cross-sectional study on younger ESRD patients revealed no significant correlation between the Montreal Cognitive Assessment (MoCA) score, used to reflect cognitive function, and diabetic ESRD patients receiving post-kidney transplant (114). In contrast, a study reported that after a 10-year follow-up, the risk of developing post-transplantation dementia in individuals with diabetic ESRD who were greater than 55 years of age significantly increased (115). Therefore, the impact of kidney transplantation on the improvement of cognitive decline in DM patients with ESRD requires further investigation.

The results of the “Tel Aviv Brain Acute Stroke Cohort” (TABASCO) study demonstrated that decreased creatinine clearance serves as an independent predictor for the development of cognitive impairment within two years after a stroke (116). Subsequent research within the TABASCO study further indicated that individuals with T2DM and reduced creatinine clearance face a higher risk of post-stroke cognitive impairment when compared to control subjects (117). These findings suggest a correlation between DKD and post-stroke cognitive impairment.

Patients with DKD frequently exhibit poor cognitive performance. A retrospective study investigated changes in cognitive function and cerebral metabolism-related indicators in DKD patients. The results revealed lower scores in abstraction and delayed recall, as assessed by the MoCA test, particularly in advanced DKD stages (97). In the Action to Control Cardiovascular Risk in Diabetes-Memory in Diabetes (ACCORD-MIND) study, which included subjects with diabetes, microalbuminuria and elevated cystatin C levels were significantly correlated with poor performance in verbal memory and executive function, respectively. High eGFR levels were not associated with improved cognitive performance (118). Moreover, another study suggested that albuminuria and low eGFR are independently linked to frontal lobe dysfunction in elderly T2DM patients, as evidenced by reduced scores on neuropsychological tests (119). Specifically, albuminuria was associated with deficits in attention and executive function, while low eGFR was linked to a decrease in psychomotor speed (119). These clinical studies indicate that markers reflecting renal dysfunction are involved in the brain-kidney connection and the pathogenesis of cognitive function in elderly individuals with chronic kidney disease and T2DM.

Considerable evidence suggests that different ethnic groups of diabetic patients exhibit varying susceptibilities to cognitive impairment. A study conducted by Anan F, et al. found that Japanese diabetic subjects with fewer cardiovascular disease risk factors (lower body mass index, shorter T2DM duration, fewer hypertensives, and fewer smokers) and preserved kidney function manifested significant correlations between microalbuminuria and cerebral WMLs (120). In African Americans (AAs), microalbuminuria showed strong correlations with several brain morphologic changes, including CSF volume and hippocampal WM volume (121). In European Americans with T2DM and mild-to-moderate renal dysfunction, kidney function parameters were not significantly associated with changes in brain structure or cognitive performance. However, non-European populations displayed contrasting results. There appear to be differential effects of renal impairment on cerebral function in T2DM individuals based on ancestry. Therefore, more research is needed to investigate the links between kidney disease, changes in cerebral structure, and cognitive performance in diverse racial groups (122).

### 5.2 Underlying mechanism of DKD-related cognitive dysfunction

Renal impairment induced by DKD is closely linked to cognitive dysfunction, which can be explained by various pathogenic mechanisms, including metabolic, vascular, and sociodemographic factors. Ghoshal et al. concluded that in patients with DKD, the presence of high albuminuria, low eGFR levels, and upregulated markers of systemic inflammation contribute to the association of nephropathy with cognitive impairment (123).

In DKD patients, inflammation activation and endothelial dysfunction are reported to be mutually interrelated, further impacting cognition (124). Hyperglycemia in DKD significantly disrupts endothelial permeability, primarily mediated by enhanced oxidative stress, inflammatory factors, and sorbitol production (94). This endothelial damage can worsen glucose metabolism disturbances in DKD patients, leading to concurrent brain dysfunction and kidney impairments due to their common vulnerabilities to endothelial injury. This is supported by findings that albuminuria develops concurrently with cerebral dysfunction (25).

Under the conditions of DKD, renal dysfunction leads to an increase in the concentration of nitric oxide inhibitors, disrupting the integrity of the BBB, which in turn contributes to cerebral vascular endothelium injury and cognitive impairment (95). Additionally, the impaired clearance of β-amyloid due to compromised renal function further contributes to the development of cognitive decline (96). These studies suggest that the impairment of clearing harmful metabolites due to poor renal function may be a potential cause of cognitive decline in the context of DKD.

Apart from its role in the high-glucose condition, NAA as a neurotransmitter which reflects neuronal damage, is significantly decreased in DKD patients, indicating heightened sensitivity of nerve cells to damage in the context of diabetic renal injury (97). Furthermore, the level of another metabolite, choline, as an integral marker of cell membrane myelin in brain tissue and a precursor of acetylcholine, is significantly elevated in DKD patients. This suggests increased cholinesterase activity, cholinergic nerve cell dysfunction, and disruptions in integrity are associated with cognitive decline in DKD patients (97).

Sonoda M et al. conducted a cross-sectional analysis and identified that low eGFR levels in patients with T2DM were associated with poor performance on the Mini-Mental State Examination (MMSE) and low plasma homocysteine concentrations (98). Interestingly, the correlation between eGFR and altered cognitive performance, as evidenced by a reduced MMSE score, could be reversed with the addition of homocysteine. This suggests that plasma homocysteine may play an indispensable and potential mediating role in connecting renal impairment with cognitive performance.

Cerebral structural lesions contribute to brain atrophy in DKD. A study demonstrated that higher eGFR levels in AAs with diabetes were associated with larger hippocampal WM volume. Conversely, higher levels of UACR, indicative of worsening proteinuria, were significantly correlated with smaller GM and hippocampal WM volume, as well as higher WML volumes, hippocampal CSF volume, and brain atrophy. Notably, no significant associations were observed between either UACR or eGFR and hippocampal GM volume (121), implying that WMLs may play a crucial role in DKD-related cognitive dysfunction. Further exploration found that for AAs with T2DM, renal apolipoprotein L1 (APOL1) risk variants were associated with decreased cerebral WML volume and larger GM volume (125), although the association between APOL1 risk variants and changes in cognitive function remains unclear.

A small-sample clinical study revealed that retinol-binding protein 4 (RBP4), a marker used to monitor DKD, may be considered a risk factor correlated with cognitive dysfunction in DKD patients with SBIs. Lipoprotein-associated phospholipaseA2 (Lp-PLA2) and Netrin-1 were also shown to be involved in the activation mechanisms of SBIs in DKD individuals, suggesting that the RBP4/Lp-PLA2/Netrin-1 signaling pathway may participate in the regulation of cognitive dysfunction in DKD patients with SBIs (99).

## 6 Pharmacological and surgical interventions to improve cognitive impairment in diabetes

At present, several potential therapeutic approaches are available to prevent and ameliorate cognitive impairments in diabetic patients. A summary of therapeutic measures alleviating cognitive dysfunction and potential mechanisms is presented in **Table 2**.

**Table 2:** The summary of drugs alleviating the cognitive decline and potential mechanisms.

Drugs	Findings	Potential mechanism	Source	Reference
ACEI and ARB	Reduction of albuminuria and decrease of cognitive decline for CKD individuals	Not applicable	ONTARGET/TRANSCEND trials	(8)
RAS blockers	Improved cognitive impairment for people with hypertension	Suppressing brain RAS activity	Cardiovascular Health Study	(126)
Telmisartan and hydrochlorothiazide	A significant preservation in cognitive function for elderly hypertensive patients	Not applicable	A prospective study	(127)
Sodium-glucose cotransporter 2 inhibitor	Improvement of brain damage	Anti-inflammation, suppression of oxidative stress, and angiogenesis and neurogenesis	The experimental studies	(128, 129)
Sodium-glucose cotransporter 2 inhibitor	A positive impact on brain health	Improving the mTOR circadian rhythm, inhibit tau pathology and the inaccurate processing of the amyloid precursor protein	Theoretical study	(130)
Empagliflozin	Preventing the impairment of cognitive function	Reducing amyloid burden in cortical regions	APP/PS1×db/db mice	(128)
Empagliflozin	Beneficial effect on cognitive function	Increase of the neurotrophic factors	db/db mice	(128)
Canagliflozin and dapagliflozin	Memory improvement	Inhibition of acetylcholinesterase	Wistar rats	(128)
Liraglutide	Diminishing the cognitive decline	Anti-inflammatory and neuroprotective properties	The experimental and observational studies	(131)
Dipeptidyl peptidase 4 inhibitor	Alleviating the cellular toxicities	Increase of neurogenesis and retention of GLP-1 function, and improvement of insulin sensitivity	Not applicable	(26, 132)
Intranasal insulin administration	Retention of impaired cerebral function	Not applicable	A pilot study	(133)
Rosiglitazone	Improvement of cognition in patients with Alzheimer’s disease	Insulin sensitizing and improvement of mitochondrial function	A perspective study	(134)
Pioglitazone	Ameliorating the cognitive decline	Activating peroxisome proliferator-activated receptors and repressing the inflammation, enhancing mitochondrial function and regulating the processing of the amyloid β peptide	A population-based longitudinal study	(135)
Antioxidants, such as creatine, vitamin E, acetyl-L-carnitine, N-acetyl cysteine, coenzyme Q10 and β-lipoic acid	Neuroprotective effect	Improvement of mitochondrial dysfunction	*In vitro*, *in vivo* and clinical studies	(136)
Intravenous sodium thiosulfate	Delaying the development of cognitive impairment	Suppressing oxidative stress and chelation with excessive metal cations, further blocking the cellular damage	Not applicable	(26)
Kidney-pancreas transplantation	Improvement of cerebral function	Not applicable	A perspective study	(93)

ACEI, angiotensin-converting enzyme inhibitor; APP/PS1, amyloid precursor protein/presenilin 1; ARB, angiotensin receptor blocker; CKD, chronic kidney disease; GLP-1, glucagon-like peptide-1; mTOR, mammalian target of rapamycin; RAS, Renin angiotensin system.

### 6.1 Proper glycemic control

Studies have shown that maintaining proper control of blood glucose can effectively ameliorate cognitive dysfunction (137, 138). Adequate glycemic control contributes to improved cognitive function in patients with T2DM (137). Conversely, the Diabetes Control and Complications Trial observed few improvements in cognitive performance in patients with T1DM (139), suggesting distinct mechanisms underlying cognitive decline in T1DM and T2DM.

### 6.2 Inhibition of the renin-angiotensin system

In general, the prevention and treatment of vascular risk factors are crucial for enhancing cognitive function. Findings from the ONTARGET/TRANSCEND trials revealed that the use of angiotensin-converting enzyme (ACE) inhibitors or ARBs contributed to the reduction of albuminuria and a decrease in cognitive decline among individuals with CKD (8). Moreover, there is ongoing investigation into the potential benefits of RAS-acting drugs on cognitive decline, considering their associations with the pathogenesis of cognitive decline (140). The Cardiovascular Health Study indicated that RAS blockers were beneficial in addressing cognitive impairment in individuals with hypertension by suppressing brain RAS activity (141). An observational open-label study also reported that the application of ARB therapy was associated with cognitive improvement (126). Additionally, a prospective study demonstrated that combination therapy involving telmisartan and hydrochlorothiazide in elderly hypertensive patients preserved cognitive function significantly (127). However, there is limited evidence supporting the protective benefits of RAS inhibitors in the cognitive dysfunction of individuals with DKD.

### 6.3 Sodium-glucose cotransporter-2 inhibitors

The therapeutic potential of SGLT2i in addressing dementia has been preliminarily explored. SGLT2i may have a neuroprotective role by suppressing acetylcholinesterase (128, 129), increasing neurotrophic factors, and improving the mammalian target of rapamycin (mTOR) circadian rhythm. They also inhibit tau pathology and the aberrant processing of the amyloid precursor protein, thus reducing the accumulation of amyloid plaques (130). The anti-inflammatory properties of SGLT2i alleviate neuronal loss caused by oxidative stress, while their inhibitory effects on angiogenesis and neurogenesis contribute to the improvement of brain damage (128). Furthermore, experimental studies have shown that SGLT2i have a beneficial effect on cognitive function. This effect may be mediated, at least in part, by an increase in neurotrophic factors and acetylcholinesterase-inhibiting activity, as well as a reduction in amyloid burden and tau pathology in the cerebral cortex. It may also be mediated by improvements in cardiovascular injury (128). Above all, the evidence suggests the potential therapeutic effect of SGLT2i on cognitive decline in diabetic patients.

### 6.4 Antioxidants

Mitochondrial dysfunction plays a crucial role in the development of neurodegenerative diseases and DC. The use of antioxidants may delay the progression of DC. Numerous studies have suggested that various antioxidants, including creatine, vitamin E, acetyl-L-carnitine, N-acetyl cysteine, coenzyme Q10, and β-lipoic acid, possess neuroprotective effects. However, the therapeutic effects have only been confirmed in only been confirmed in small clinical studies, and larger clinical trials are required to investigate their potential benefits and mechanisms (136).

### 6.5 Kidney transplantation

The impact of kidney transplantation on the improvement of cognitive decline in diabetic ESRD patients remains controversial. For ESRD patients, kidney-pancreas transplantation is a viable approach to control hyperglycemia and delay the progression of diabetic nephropathy and encephalopathy (142). Remarkable improvements in cerebral function have been observed with kidney-pancreas transplantation (93). However, an observational study reported a dramatic increase in the risk of post-renal transplant dementia after a 10-year follow-up (115). Therefore, it is essential to carefully assess the effects of kidney transplantation on cognitive function.

### 6.6 Other novel therapies

Intravenous sodium thiosulfate (STS) is a novel and effective antioxidative therapy for delaying the development of cognitive impairment. It may be beneficial by chelating excessive metal cations and blocking cellular damage in the brain caused by abnormal interactions of metal ions with Aβ and oxidative stress in the development of DC (26).

The glucagon-like peptide 1 (GLP-1) receptor is widely expressed in key brain regions, including the hypothalamus, hippocampus, and cerebral cortex (143). Experimental studies have demonstrated that GLP-1-positive cells and proglucagon mRNA, which play a role in mediating obesity and insulin resistance, were significantly reduced in the hippocampus and cortex of ob/ob mice compared to control mice (143). Moreover, microglial cells (MGCs), a primary source of GLP-1 secretion in the brain, have been implicated in early neurodegeneration in diabetic mouse models (144), highlighting the GLP-1-mediated impact on cognitive function. Notably, liraglutide, a GLP-1 receptor agonist, has been shown to exhibit neuroprotective effects and mitigate cognitive decline in both experimental and observational studies (131). The anti-inflammatory and neuroprotective properties of GLP-1 agonists make them promising candidates for treating cognitive decline. Additionally, dipeptidyl peptidase 4 (DPP-4) inhibitors, commonly used oral hypoglycemic agents, have been reported to alleviate potential cellular toxicities by increasing neurogenesis and preserving GLP-1 function in diabetic DC patients (26, 132). However, more robust clinical trials are needed to confirm the precise therapeutic effects of DPP-4 inhibitors on cognitive decline.

Insulin resistance in the brain is a significant factor in the development of DC. Small pilot studies have indicated that intranasal insulin administration is a feasible treatment modality that can enhance cognitive functions, including delayed verbal memory and attention (133), offering a potential approach for addressing neurodegenerative disorders and cognitive decline. Furthermore, insulin-sensitizing agents, which are classic treatments for improving glycemic control, have shown promise in enhancing cognition. Studies suggest that rosiglitazone, an insulin-sensitizing drug, may have potential cognitive benefits in patients with Alzheimer’s disease (134). However, the therapeutic effects of rosiglitazone on cognitive decline in DKD patients require further investigation. Additionally, pioglitazone, another insulin-sensitizing drug that exerts its glucose-lowering effects through the activation of peroxisome proliferator-activated receptors, has also been reported to ameliorate cognitive decline. Nevertheless, more clinical research is necessary to fully understand its impact (135).

**Figure 4:**
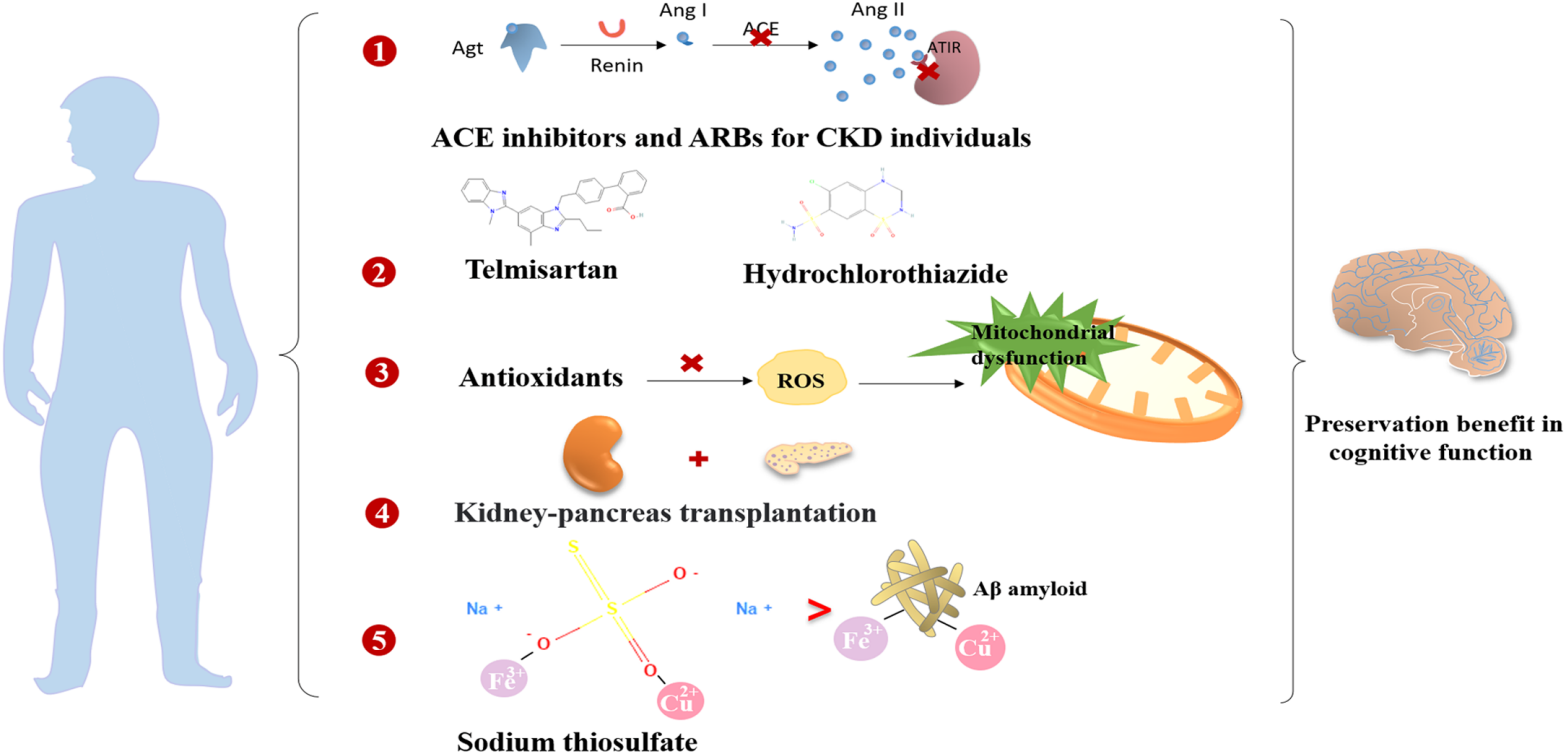
The preservation benefits of non-hypoglycemic drugs and therapeutic measures on the cognitive function.

**Figure 5:**
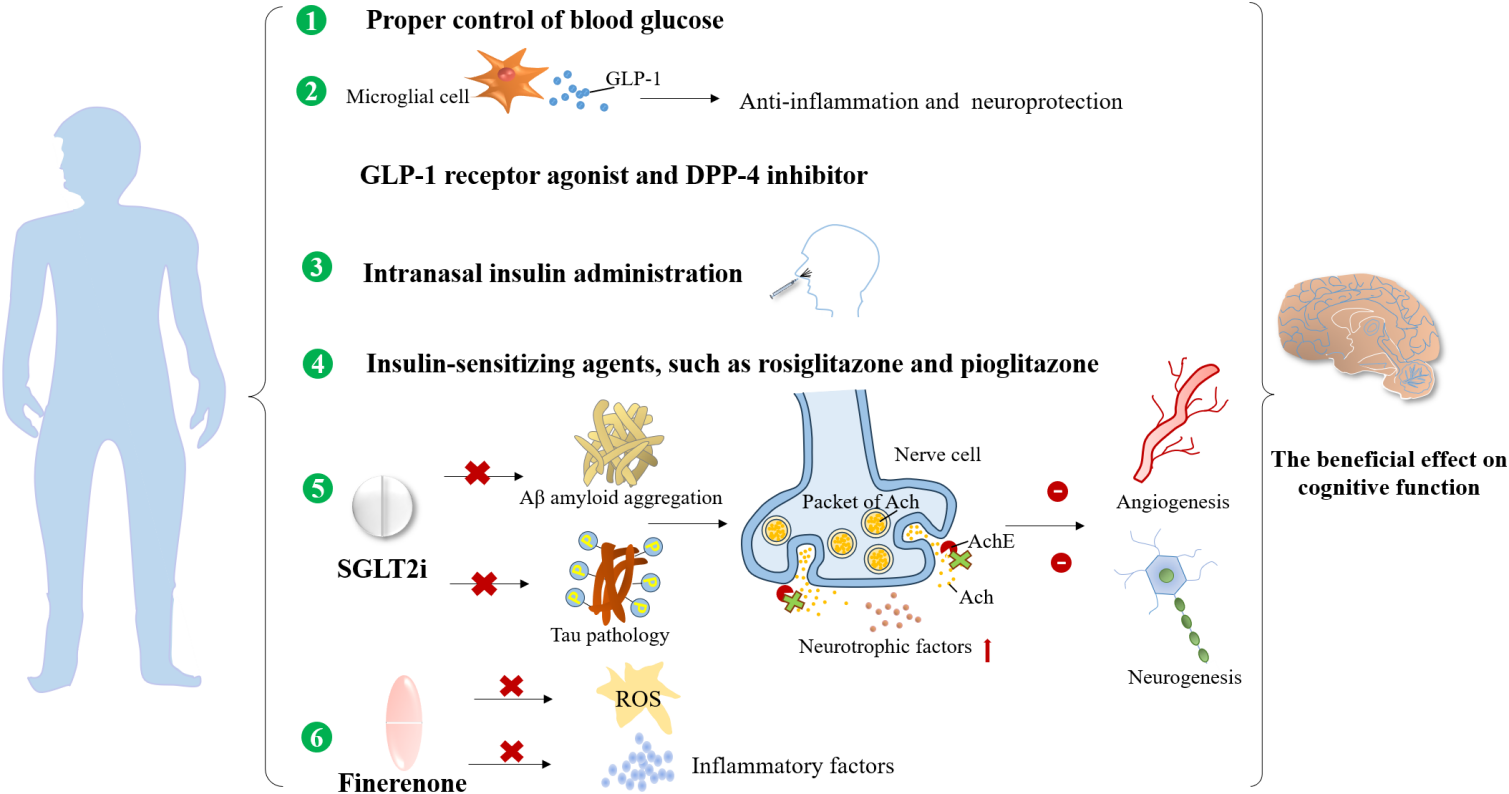
The beneficial effects of anti-hyperglycemic drugs on the cognitive dysfunction.

Finerenone, a novel selective mineralocorticoid receptor antagonist (MRA), has manifested both cardiovascular and kidney protection confirmed by two phase III trials, Finerenone in Reducing Kidney Failure and Disease Progression in Diabetic Kidney Disease (FIDELIO-DKD) (145) and Finerenone in Reducing Cardiovascular Mortality and Morbidity in Diabetic Kidney Disease (FIGARO-DKD) (146) together with FIDELITY pooled analysis (147). There are substantial experimental studies demonstrating that finerenone ameliorated endothelial impairment through upregulation of nitric oxide bioavailability and inhibition in ROS (148) and inflammatory factors, such as IL-6 and transforming growth factor-β (TGF-β) in the kidney (149). Considering that kidney and brain dysfunction share common pathogenic factors, such as vascular endothelial injury, oxidative stress, and inflammation activation, we speculate that finerenone may achieve the cognitive preservation by suppressing the production of ROS and pro-inflammatory cytokines, and could improve cerebral microvascular function by alleviating endothelial dysfunction. However, dedicated clinical studies in diabetic and DKD populations are needed to explore the potential cognitive improvement effects of finerenone for DKD patients in future. A summary of the cognitive preservation benefits of non-hypoglycemic and anti-hyperglycemic drugs is provided in **Figures 4** and **5**.

## 7 Conclusion

Understanding the mechanisms of crosstalk of the kidney and brain may offer insights into preventing cognitive impairment in T2DM populations. Early recognition and prediction of diabetes-related cognitive impairment can be valuable in halting the progression of dementia and related detriments. In summary, future studies on diabetes should concentrate on exploring the influence of abnormal renal function as mediators of cerebral impairment to better comprehend the mechanisms of the kidney-brain axis.

## Glossary

AAs: African Americans
ACCORD-MIND: Action to Control Cardiovascular Risk in Diabetes-Memory in Diabetes
ACE: angiotensin-converting enzyme
AGE: advanced glycation end product
Agt: angiotensinogen
Ang I: angiotensin I
Ang II: angiotensin II
APOL1: apolipoprotein L1
APP/PS1: amyloid precursor protein/presenilin 1
ARB: angiotensin receptor blocker
ARIC: Atherosclerosis Risk in Communities
AT1R: angiotensin II type 1 receptor
BBB: blood-brain barrier
BRINK: Brain in Kidney Disease
Cho: choline
CKD: chronic kidney disease
CRP: C-reactive protein
CSF: cerebrospinal fluid
cSVD: cerebral small vessel disease
CX3CR1: C-X3-C motif chemokine receptor 1
DC: diabetic cognopathy
DCCT: Diabetes Control and Complications Trial
DKD: diabetic kidney disease
DM: diabetes mellitus
DPP-4: dipeptidyl peptidase 4
eGFR: estimated glomerular filtration rate
ESRD: end-stage renal disease
FGF-23: fibroblast growth factor 23
FIDELIO-DKD: Finerenone in Reducing Kidney Failure and Disease Progression in Diabetic Kidney Disease
FIGARO-DKD: Finerenone in Reducing Cardiovascular Mortality and Morbidity in Diabetic Kidney Disease
GLP-1: glucagon-like peptide 1
GM: gray matter
HbA1c: glycosylated hemoglobin
IL-1β: interleukin-1β
IL-6: interleukin-6
INVADE: Intervention Project on Cerebrovascular Diseases and Dementia in the community of Ebersberg
LADIS: Leukoaraiosis and Disability Study
Lp-PLA2: lipoprotein-associated phospholipase A2
MGCs: microglial cells
MMSE: Mini-Mental State Examination
MoCA: Montreal Cognitive Assessment
MRA: mineralocorticoid receptor antagonist
MRI: magnetic resonance imaging
mTOR: mammalian target of rapamycin
NAA: N-acetyl aspartate
NAME: Nutrition, Aging, and Memory in Elders
NHANES III: Third National Health and Nutrition Examination Survey
Nrf2: nuclear factor erythroid 2-related factor 2
RAAS: renin-angiotensin-aldosterone system
RAGE: receptor for AGE
RAS: renin-angiotensin system
RBP4: retinol-binding protein 4
REGARDS: Reasons for Geographic and Racial Differences in Stroke
ROS: reactive oxygen species
SBIs: silent brain infarcts
SGLT2i: sodium-glucose cotransporter-2 inhibitor
STS: sodium thiosulfate
TABASCO: Tel Aviv Brain Acute Stroke Cohort
T1DM: type 1 diabetes mellitus
T2DM: type 2 diabetes mellitus
TGF-β: transforming growth factor-β
TNF-α: tumor necrosis factor-α
UACR: urine albumin-to-creatinine ratio
WMH: white matter hyperintensities
WMLs: white matter lesions.
